# Toll-like receptor ligands induce the expression of interferon-gamma and interleukin-17 in chicken CD4+ T cells

**DOI:** 10.1186/1756-0500-5-616

**Published:** 2012-11-01

**Authors:** Michael St Paul, Neda Barjesteh, Sarah Paolucci, Yanlong Pei, Shayan Sharif

**Affiliations:** 1 Department of Pathobiology, Ontario Veterinary College, University of Guelph, Guelph, Ontario, N1G 2W1, Canada

**Keywords:** Chicken, T cell, Toll-like receptor, Interferon-γ, Interleukin-17, CD4+ T-helper, Pam3CSK4, Poly I:C, LPS, CpG

## Abstract

**Background:**

Toll-like receptors (TLRs) are evolutionarily conserved pattern recognition receptors that mediate host responses to pathogens. To date, at least 10 different TLRs have been identified in chickens including TLR2, which binds lipopeptides and other similar ligands such as Pam3CSK4, TLR3, which binds double stranded RNA as well as synthetic molecules such as poly I:C, TLR4, which binds lipopolysaccharide (LPS), and TLR21, which binds CpG DNA motifs. In mammals, TLRs have been detected on CD4+ T cells where they mediate cellular survival, proliferation and the production of cytokines. However, the TLR-mediated responses in chicken CD4+ T cells remain to be determined. As such, the objective of the present study was to elucidate the kinetics of cytokine response to several different TLR ligands in chicken CD4+ T cells.

**Results:**

The results suggest that these cells express TLRs 2, 3, 4 and 21 at the transcript level, and treatment with ligands for these TLRs significantly influenced the expression of the cytokines interferon (IFN)-γ and interleukin (IL)-17, but not IL-4, IL-10 and IL-13. Specifically, treatment with Pam3CSK4, poly I:C and LPS up-regulated IFN-γ transcripts, while CpG ODN significantly down-regulated them. In contrast, at least one dose of each of the TLR ligands, except for Pam3CSK4, significantly down-regulated IL-17 transcripts.

**Conclusion:**

Chicken CD4+ T cells respond to ligands for TLRs 2, 3, 4 and 21 by up-regulating or down-regulating cytokine transcripts. Future studies may consider exploring how these TLR ligands may modulate other effector functions in chicken CD4+ T cells, as well as in other T cell subsets such as CD8+ T cells.

## Background

Toll-like receptors (TLRs) are evolutionarily conserved pattern recognition receptors that bind conserved motifs on pathogens termed pathogen associated molecular patterns (PAMPs) [1]. To date, at least 10 different TLRs have been identified in chickens including TLR2, which binds lipopeptides (e.g. Pam3CSK4) and other similar ligands, TLR3, which binds double stranded RNA (e.g. poly I:C), TLR4, which binds lipopolysaccharide (LPS), and TLR21, which binds CpG DNA motifs [2]. Toll-like receptors have been detected in several cell subsets including in macrophages, heterophils and B cells [3].

Interactions between TLRs and their ligands typically results in cellular activation, enhanced effector functions and the production of cytokines. In the case of mammalian T cells, TLR stimulation promotes cell proliferation and survival as well as induction of cytokines, such as interferon (IFN)-γ [4-7]. In addition, TLR stimulation may also promote the differentiation of naïve CD4+ T cells into one of the many different T-helper (T_H_) cell subsets [8]. To date, several different TLRs have been detected in mammalian CD4+ T cells [9].

Although well documented in mammals, the TLR-mediated responses in chicken T cells have yet to be elucidated. So far, a few TLRs have been identified in chicken CD4+ T cells including TLRs 2, 3 and 4 [3]. However, the full repertoire of TLRs expressed in chicken CD4+ T cells and their responses to TLR ligands have yet to be characterized. To this end we hypothesized that chicken T cells express TLRs and respond to TLR ligands by up-regulating cytokine transcripts. As such, the objective of the present study was to examine the kinetics of the TLR-mediated cytokine response in chicken CD4+ T cells. The results suggest that CD4+ T cells express TLRs 2, 3, 4 and 21 at the transcript level, and treatment with ligands for these TLRs significantly influenced the expression of the cytokines IFN-γ and interleukin (IL)-17, but not IL-4, IL-10 and IL-13.

## Results and discussion

Toll-like receptor ligands have previously been shown to modulate the production of cytokines in several chicken cell subsets, including in macrophages, heterophils and B cells [10-12]. Here, we show that a similar phenomenon may be extended to chicken CD4+ T cells.

**Figure 1:**
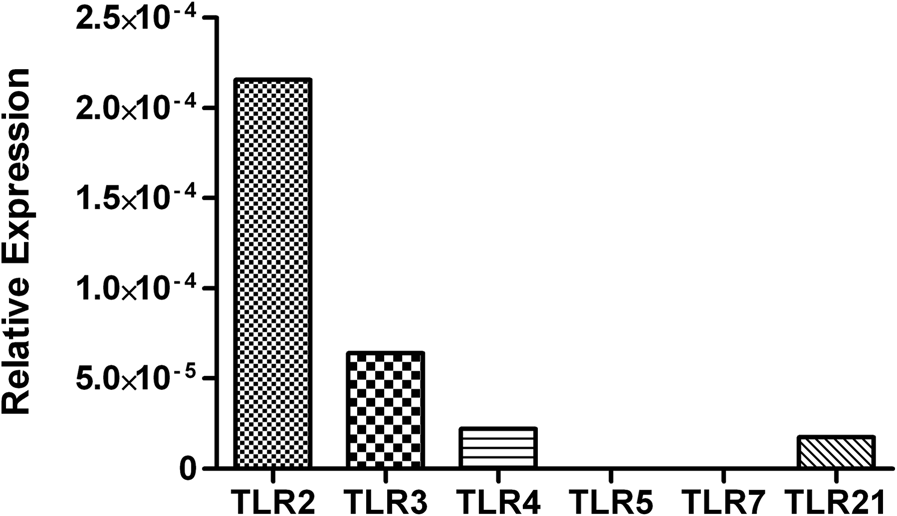
**TLR expression in chicken CD4+ T cells.** Gene expression of TLR transcripts in un-stimulated chicken CD4+ T cells relative to the house-keeping gene β-actin.

In mammals, CD4+ T cells may be classified into several different subsets such as T_H_1, T_H_2, T_H_17 and regulatory T cells (T_REG_) [13]. In addition to producing a distinct profile of cytokines and performing different effector functions, each cell subset also expresses a different repertoire of TLRs. For example, TLR10 has been detected in human regulatory T cells, but not in non-regulatory CD4+ T cells [14]. Nevertheless, CD4+ T cells, in general, express transcripts for TLRs 2, 3, 4, 5, 7/8 and 9 in both mice and humans [9]. In chicken CD4+ T cells, it was shown that they also express TLRs transcripts including those for TLRs 2, 3 and 4 [3]. However, the study by Iqbal et al., (2005) used semi-quantitative PCR and as such, we employed real-time PCR to provide a more accurate quantification of TLR transcript levels (Figure 1). Our results suggest chicken CD4+ T cells express TLRs 2, 3, 4 and 21 at the transcript level, and not TLRs 5 and 7. Moreover, transcripts for TLR2 were the most abundant, followed by transcripts for TLR3 and lastly by TLRs 4 and 21. This therefore raises the possibility that chicken CD4+ T cells may have the potential to respond directly to PAMPs derived from both viral and bacterial pathogens.

**Figure 2:**
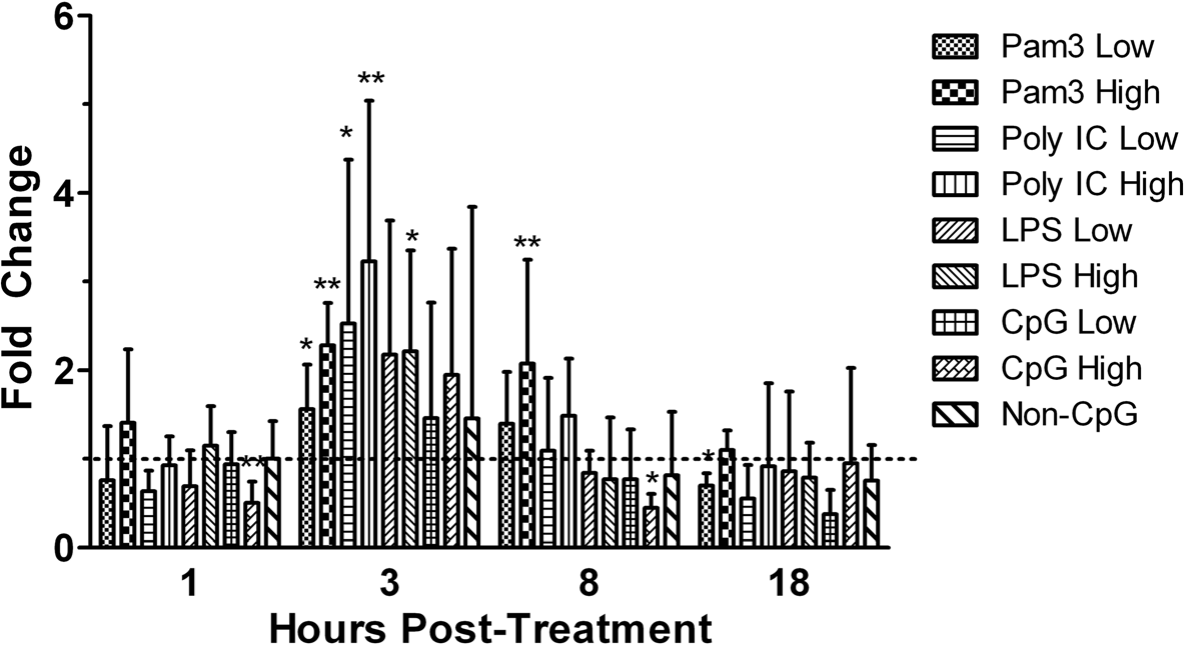
**Gene expression of IFN-γ.** Relative gene expression of IFN-γ in chicken CD4+ T cells at 1, 3, 8 and 18 hours post-treatment with low (1 μg/mL) and high (5 μg/mL) doses of the TLR ligands Pam3CSK4, LPS and CpG ODN, or with a low (10 μg/mL) or high (50 μg/mL) dose of poly I: C. Graphed data represent mean fold change of 4 treatment replicates compared to the medium control group ± standard error. Results were considered statistically significant from the medium-treated control group if p ≤ 0.05 (*) and p ≤ 0.01 (**).

**Figure 3:**
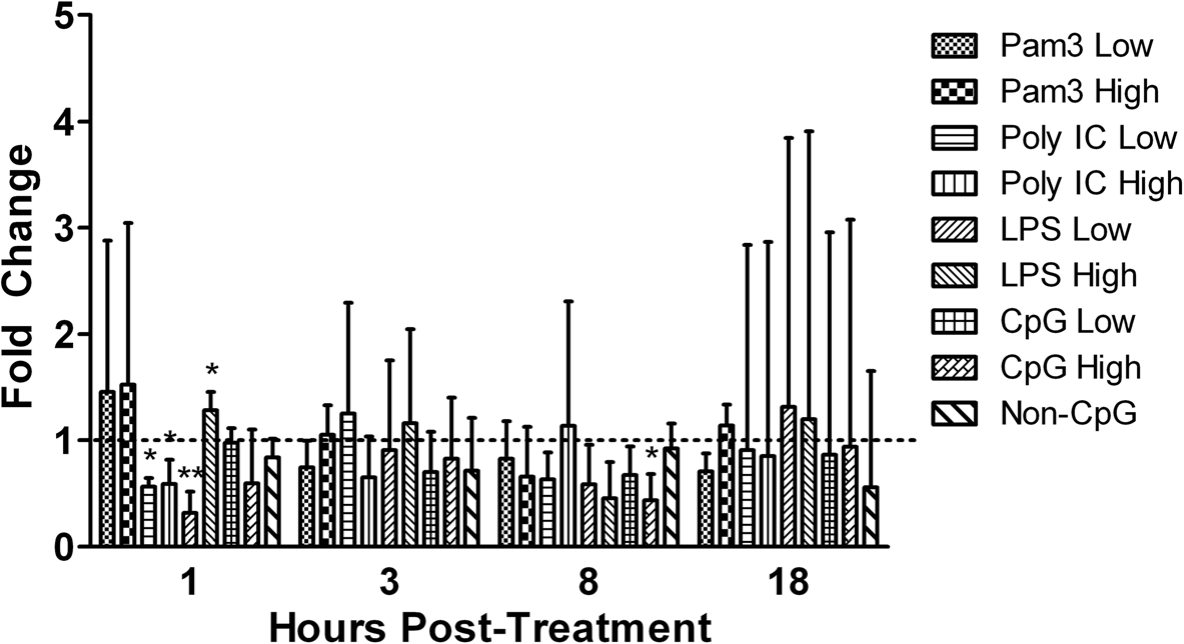
**Gene expression of IL-17.** Relative gene expression of IL-17 in chicken CD4+ T cells at 1, 3, 8 and 18 hours post-treatment with low (1 μg/mL) and high (5 μg/mL) doses of the TLR ligands Pam3CSK4, LPS and CpG ODN, or with a low (10 μg/mL) or high (50 μg/mL) dose of poly I:C. Graphed data represent mean fold change of 4 treatment replicates compared to the medium control group ± standard error. Results were considered statistically significant from the medium-treated control group if p ≤ 0.05 (*) and p ≤ 0.01 (**).

Different mammalian CD4+ cell subsets produce a distinct profile of cytokines upon stimulation. These cytokines include IFN-γ, which is produced by T_H_1 cells and IL-4/13 which are produced by T_H_2 cells, as well as IL-17 and IL-10, which are produced by T_H_17 cells and T_REGS_, respectively. Although in chickens it is not yet known if such CD4+ T cell subsets exist, evidence accumulated over the last few years raises the possibility that at least some of these subsets might [15-17]. As such, in the present study, we examined the above cytokines to determine how TLR ligands modulate their expression (Figures 2 and 3).

In mammals, TLR2 ligands such as Pam3CSK4 have been shown to directly activate CD4+ T cells and induce their production of IFN-γ, in the absence of T cell receptor (TCR) signaling [5,7]. Our results suggest that this may also be the case in chicken CD4+ T cells, as treatment with Pam3CSK4 significantly up-regulated IFN-γ transcripts at 3 (p ≤0.01) and 8 (p ≤0.01) hours post-treatment (Figure 2). This effect was not limited only to Pam3CSK4, as both poly I:C (p ≤0.01) and LPS (p ≤0.05) significantly enhanced IFN-γ transcripts levels as well (Figure 2). This is in contrast to what occurs in mammals, as poly I:C and LPS fail to directly up-regulate IFN-γ production in T_H_1 cells [7]. In fact, when combined with TCR stimulation, LPS inhibits IFN-γ production in mammalian T cells, which was shown to be mediated by the TIR-domain-containing adapter-inducing interferon-β (TRIF) pathway [18]. In chickens, emerging evidence suggests that both TLRs 3 and 4 signal through the TRIF pathway as indicated by a robust type I IFN response following treatment with these ligands [19-21]. However, as these ligands both up-regulated IFN-γ transcripts in the present study, this raises the possibility that there might be some differences between the chicken and mammalian TRIF pathways, potentially with respect to the accessory and signaling molecules involved. As such, future studies should be aimed at further elucidating the mechanisms involved in the TRIF signaling pathway in chickens.

We discovered that there was a significant down-regulation (controls were set to 1) of IFN-γ transcripts following treatment CpG ODN at 1 (p ≤0.01) and 8 (p ≤0.05) hours post-treatment (Figure 2). This is in contrast with what has been shown in mammalian CD4+ T cells, because CpG ODN enhances production of cytokines such as IL-2 and IFN-γ by these cells [22]. However, this enhanced cytokine production occurs only in conjunction with TCR signaling, and as such, future studies may consider exploring whether adding anti-chicken CD3 may alter the responses to CpG ODN and other TLR ligands.

In addition to enhancing IFN-γ production, TLR ligands may also modulate responses of other T cell subsets, such as T_H_17 cells. For example, treatment of naïve CD4+ T cells with Pam3CSK4 promotes their differentiation into T_H_17 cells [8]. Importantly, when fully differentiated T_H_17 cells are treated with TLR ligands including Pam3CSK4 and LPS, but not poly I:C, a significant increase in IL-17 production is observed [8]. However, this does not appear to be the case in chickens, as suggested by our results (Figure 3). We found that both poly I:C, and the high dose of CpG ODN (p ≤0.01) significantly down-regulated IL-17 transcripts at 1 hour and 8 hours post-treatment, respectively. Moreover, we found that treatment with the low dose of LPS significantly down-regulated IL-17 transcripts at 1 hour post-treatment (p ≤0.01), while the high dose significantly up-regulated IL-17 at 1 hour post-treatment (p ≤0.05). Although the reason behind this observation is not known, we have observed a similar down-regulation of murine natural killer T (NKT) cell activities in response to some TLR ligands, including CpG ODN, which we have attributed to a TLR-mediated increase in dual specific protein phosphatases (DUSPs) (Villanueva et al., unpublished data). As such, future studies may consider employing additional assays in order to examine the role of DUSPs in chicken TLR mediated responses.

In mammals, evidence suggests that T_H_2 cells are non-responsive to TLR ligands and fail to become activated and up-regulate the production of IL-4 [7]. This seems to also be the case in chickens, as we did not detect any significant up-regulation of IL-4 or IL-13 in response to any of the TLR ligand treatments (data not shown). In addition, we also did not detect any significant up-regulation of IL-10 either in response to Pam3CSK4 or any of the other TLR ligands (data not shown). Although in mammals IL-10 may be produced by T_H_2 cells as well as T_REGS_, in chickens, evidence suggests that stimulated CD4+ CD25+ regulatory T cells are the predominant source of IL-10, as they produce more than 30 times the amount of IL-10 when compared against stimulated CD4+ CD25- T cells [15]. Nevertheless, TLR ligands have been shown to directly activate mammalian T_REGS_ and promote their proliferation and survival, however this occurred only in conjunction with TCR stimulation [4]. Therefore, we speculate that this lack of up-regulation in the chicken CD4+ T cells may be due to lack of TCR stimulation. However, there are a few other possible explanations that could be considered. For example, i) chicken T_REGS_ may not respond to TLR ligands or ii) our T cell population may potentially be limited in its diversity and may have an oligoclonal or monoclonal nature. As a result, the population of T cells that we have used in the present study might have been devoid of T_H2_ and T_REG_ populations.

## Conclusions

In conclusion, we have shown that chicken CD4+ T cells express several TLRs at the transcript level and respond to treatment with TLR ligands by modulating the expression of IFN-γ and IL-17 transcripts, but not IL-4, IL-10 or IL-13. Future studies may consider exploring how these TLR ligands may modulate other effector functions in chicken CD4+ T cells, as well as in other T cell subsets such as CD8+ T cells.

## Methods

### TLR ligands

Pam3CSK4 was purchased from Invivogen (Burlington, ON), poly I:C and LPS from *Escherichia coli* 0111:B4 were purchased from Sigma-Aldrich-Canada (Oakville, ON), while synthetic class B CpG ODN 2007 [5’- TCGTCGTTGTCGTTTTGTCGTT-3’] and non-CpG ODN [5’- TGCTGCTTGTGCTTTTGTGCTT-3’] were purchased from Eurofins MWG Operon (Ebersberg, GER). All of the ligands used were re-suspended in sterile water or phosphate buffered saline (PBS, pH 7.4) and diluted to working concentrations in complete RPMI medium.

### Stimulation of T cells with TLR ligands

Reticuloendotheliosis virus transformed CD4+ T cells were generated using the protocol previously described for transforming chicken B cells [23], with slight modifications. In the present study, mononuclear splenocytes from 1 week-old chickens were used as the starting cell population. After transformation, the cells were passaged several times, leading to elimination of non-transformed cells. Subsequently, purity of the transformed cells was assessed using flow cytometry. T cells were then cultured in RPMI-1640 (Invitrogen, Burlington, ON) supplemented with 10% heat-inactivated fetal bovine serum, 200 U/mL penicillin, 80 μg/mL streptomycin, 25 mg gentamicin, 10 mM HEPES buffer, 50 μM β-mercaptoethanol, and 2 mM L-glutamine, and seeded into 48-well plates at 1x10^7^ cells/mL for *in vitro* stimulation with either a low (1 μg/mL) or high (5 μg/mL) dose of each TLR ligand, except for poly I:C, which was delivered at 10 μg/mL or 50 μg/mL, respectively. The control groups received non-CpG ODN (5 μg/mL) or medium. Cells were harvested at 1, 3, 8 and 18 hours post-stimulation for RNA extraction.

### RNA extraction and cDNA synthesis

Total RNA was extracted from T cells using TRIzol® (Invitrogen, Carlsbad, CA) according to the manufacturer’s protocol and treated with DNA Free® (Ambion, Austin, TX) DNAse. Subsequently, 1μg of purified RNA was reverse transcribed to cDNA using Superscript® II First Strand Synthesis kit (Invitrogen, Carlsbad, CA) and oligo-dT primers, according to the manufacturers recommended protocol. The resulting cDNA was subsequently diluted 1:10 in DEPC treated water.

### Real-time PCR

Quantitative real-time PCR using SYBR Green was performed on diluted cDNA using the LightCycler® 480 II (Roche Diagnostics GmbH, Mannheim, GER) as previously described [19]. Briefly, each reaction involved a pre-incubation at 95°C for 10 min, followed by 45 cycles of 95°C for 10 min, 55°C −64°C (T_A_ as per primer) for 5 s, and elongation at 72°C for 10 s. Subsequent melt curve analysis was performed by heating to 95°C for 10 s, cooling to 65°C for 1 min, and heating to 97°C. Primers were synthesized by Sigma-Aldrich-Canada (Oakville, ON), and their specific sequences and accession numbers are outlined in Table 1.

**Table 1:** Primer sequences and accession numbers used for real-time PCR

**Target gene**	**Primer sequence**	**Accession number**
TLR2	F: 5’- ATCCTGCTGGAGCCCATTCAGAG -3’	[GenBank: NM_204278.1/NM_001161650]
	R: 5’- TTGCTCTTCATCAGGAGGCCACTC -3’	
TLR3	F: 5’- TCAGTACATTTGTAACACCCCGCC -3’	[GenBank: DQ780341]
	R: 5’- GGCGTCATAATCAAACACTCC -3’	
TLR4	F: 5’- TGCCATCCCAACCCAACCACAG -3’	[GenBank: AY064697.1]
	R: 5’- ACACCCACTGAGCAGCACCAA -3’	
TLR5	F: 5’- TTCTTGCAACCTCACAGGTGTTCC -3’	[GenBank: NM_001024586.1]
	R: 5’- CAGGTCCAAGACACGAAGATT -3’	
TLR7	F: 5’- TTCTGGCCACAGATGTGACC -3’	[GenBank: NM_001011688]
	R: 5’- CCTTCAACTTGGCAGTGCAG -3’	
TLR21	F: 5’- CCTGCGCAAGTGTCCGCTCA -3’	[GenBank: AJ720600.1]
	R: 5’- GCCCCAGGTCCAGGAAGCAG -3’	
IFN-γ	F: 5’- ACACTGACAAGTCAAAGCCGCACA-3’	[GenBank: X99774]
	R: 5’-AGTCGTTCATCGGGAGCTTGGC-3’	
IL-4	F: 5’-TGTGCCCACGCTGTGCTTACA-3’	[GenBank: AJ621249.1]
	R: 5’- CTTGTGGCAGTGCTGGCTCTCC-3’	
IL-10	F: 5’- AGCAGATCAAGGAGACGTTC −3’	[GenBank: AJ621614]
	R: 5’- ATCAGCAGGTACTCCTCGAT −3	
IL-13	F: 5’- ACTTGTCCAAGCTGAAGCTGTC -3’	[GenBank: AJ621250]
	R: 5’- TCTTGCAGTCGGTCATGTTGTC -3’	
IL-17	F: 5’- CACTGCTGTTGGTGTTGCT -3’	[GenBank: AJ493595]
	R: 5’- TCAGCAACCAAGCGGGGG -3’	
β-Actin	F: 5’-CAACACAGTGCTGTCTGGTGGTA-3’	[GenBank: X00182]
	R: 5’-ATCGTACTCCTGCTTGCTGATCC-3’	

### Data analysis

Relative expression levels of all genes was calculated relative to the housekeeping gene β-actin using the LightCycler® 480 Software (Roche Diagnostics GmbH, Mannheim, GER), based on the formula developed by Pfaffl [24]. Data represent mean fold change of 4 replicates compared to the medium control group ± standard error. The transcript levels in the medium-treated control group were set to 1. Results were considered statistically significant from the medium-control group if p ≤ 0.05 (*) and p ≤ 0.01 (**). Fold changes, standard error and statistical significance were calculated using the software REST 2009 (Qiagen, Valencia, CA) [25].

## Abbreviations

TLR: Toll-like receptor; LPS: Lipopolysaccharide; ODN: Oligodeoxynucleotides; IFN: Interferon; IL: Interleukin; CD: Cluster of differentiation; PAMPs: Pathogen associated molecular patterns; T_H_: T-helper; TCR: T cell receptor; NKT: Natural killer T cell; DUSPs: Dual specific protein phosphatases.

## Competing interests

The authors declare that they have no competing interests.

## Authors' contributions

MSP designed and conducted the experiment, processed the samples, analyzed the results and drafted the manuscript. NB, SP and YP assisted with conducting the experiment, processing the samples and revising the manuscript. SS assisted with designing the experiment and drafting and revising the manuscript. All authors have read and approved the final manuscript.
